# Mutual Involvement in Families With Type 2 Diabetes Through Web-Based Health Care Solutions: Quantitative Survey Study of Family Preferences, Challenges, and Potentials

**DOI:** 10.2196/diabetes.7456

**Published:** 2017-09-27

**Authors:** Tobias Vitger, Henning Langberg, Dan Grabowski

**Affiliations:** 1 Health Promotion Research Steno Diabetes Center Copenhagen Gentofte Denmark; 2 CopenRehab Department of Public Health, Section of Social Medicine, Faculty of Health and Medical Sciences University of Copenhagen Copenhagen Denmark

**Keywords:** online, health information, diabetes, support, family

## Abstract

**Background:**

Type 2 diabetes (T2D) is a prevalent chronic disease that affects not just patients but entire families. Both the patient and the rest of the family may benefit from gaining knowledge about the disease and from supportive interfamilial interaction. The Internet is becoming a widely-used resource for health information, so a Web-based solution could potentially promote awareness and knowledge on how to manage T2D as a family, while also providing support for the family.

**Objective:**

We aim to assess the usage of online diabetes information by patients with T2D and their relatives, and explore the families’ needs and preferences regarding online information on diabetes.

**Methods:**

A quantitative self-reported questionnaire survey was performed with Danish families that had at least one family member diagnosed with T2D. The survey consisted of 36 closed questions on demographics, usage of the Internet, preferences in the source of information, interest in online information on six problem domains within family life related to T2D, preferences towards the delivery format of online information, and peer-to-peer communication. Two open-ended questions were also included to elicit any additional comments or suggestions about improving online information on T2D regarding family life.

**Results:**

Fifty participants from 22 families with T2D answered the questionnaire individually. Relatives (25/28, 89%) and patients (22/22, 100%) indicated that information on T2D is relevant for them, while indicating that the Internet is the first or second preferred source when in need of information on T2D (25/28, 89% vs 21/22, 95%). Only a minority of the participants indicated that they had searched the Internet to gain knowledge on T2D regarding family life (9/28, 32% vs 10/22, 46%). Also, patients were more likely to have used the Internet to gain information on T2D (*P*=.027). Both groups indicated a preference for watching videos or reading about T2D in relation to family life while a minority of the participants indicated an interest in peer-to-peer communication. Regarding the six problem domains, the domains Support, Knowledge, and Everyday Life were slightly more popular. These three domains were considered interesting by at least 79% (22/28) and 73% (16/22) of the relatives and patients respectively, while the domains Communication, Worries, and Roles were considered interesting by at least 46% (20/28) and 50% (11/22).

**Conclusions:**

Despite an interest in online information on T2D, there appears to be an unsatisfied need for more supportive online information on T2D aimed at Danish families with T2D. Based on family preferences, online information should focus on the six problem domains and be presented through text and videos by health care practitioners and peers. Peer-to-peer communication elements may be beneficial, but are only expected to be used by a very limited number of families.

## Introduction

Type 2 diabetes (T2D) affects more than 300 million people worldwide and projections indicate that more than 1.1 billion people will be either living with diabetes or at high risk of diabetes in 2040 [1]. Most patients with T2D provide more than 90% of their own daily care, so health behavior interventions often seek to improve the patient’s lifestyle, medication adherence, and diabetes management [2]. Many of these interventions have found social support, such as the involvement of the patient’s relatives (eg, spouses, family, offspring, close friends) to be an effective means of improving the patient’s health behavior or self-care [3]. With the rapid development of the World Wide Web, the Internet can facilitate supportive interaction while also being an increasingly popular method to gain educational information on diabetes [4-6].

Social support is an important positive factor related to the patient’s diabetes management, either by facilitating healthy behaviors (eg, buying or preparing healthy meals) or by helping the patient overcome stress and frustrations through communication [3]. Contrarily, relatives can also have a negative impact on the patient’s diabetes management by representing a barrier (rather than a facilitator) towards healthy behaviors, or by being supportive in an unappreciative way [3]. Relatives who are living in the same household as a patient with T2D may also be affected by the patients’ disease through changes in the family’s everyday life and family roles, while also being at increased risk of developing T2D, mainly due to genetics, lifestyle, and a lack of awareness about this risk [7,8]. Promoting mutual involvement between patients and relatives is often met by multiple barriers such as: families not perceiving the relevance of including relatives in the management of T2D, individuals who are at risk of developing T2D being apparently less engaged in risk-reducing health behavior, and patients with T2D seldom expressing serious concerns about relatives developing T2D [9-12]. Despite the seemingly important factor of social support in T2D, few intervention studies on T2D management have included the whole family (ie, the patient and their relatives) [3]. A study by Zrebiec and Jacobson found that an online discussion group on different diabetes topics moderated by health care providers (HCPs) was a useful method to engage both patients and relatives in receiving emotional support and exchanging information [4]. However, most diabetes websites do not provide sufficient information to support patients, making it unlikely that the information is sufficient for the rest of the family [6,13]. Web-based interventions for other chronic diseases have found improvements in interfamilial communication, knowledge on managing symptoms, medication adherence for the patients, and reduced stress levels for relatives in the role of caregivers [14-17]. Online information has also been assessed as a useful supplement to the information gained from consultations with HCPs [18].

Although the literature suggests that Web-based solutions could be a promising tool for families with T2D, research on the whole family’s preferences regarding online information aimed at families with T2D is lacking. Previous studies investigating online health information on T2D have primarily focused on the patient, and assessments of the information aimed at supporting the whole family are needed, together with more evidence on the effects of Web-based health care solutions for families with T2D. Hillard et al argued that there is a need for research to better understand both patients’ and relatives’ reasons for, or their barriers to, participating in diabetes online communities [19]. More research on family preferences and their needs related to information on T2D is needed to develop more personalized (and potentially more effective) Web-based health care solutions for the whole family. In addition to the content of online information, several studies highlight the need for presenting relevant information in an understandable and compelling format to the end-user; a focus that is often left out in scientific research [20,21].

In a previously reported comprehensive qualitative study, we investigated problems and challenges associated with family life in families with T2D [22]. We described six problem domains: *Support*, *Knowledge*, *Communication*, *Worries*, *Roles*, and *Everyday Life* [22]. The study described here serves as an extension of this qualitative study, and will provide quantitative data on families’ interests and preferences in terms of online information. The main objective of this study was to use these six problem domains to provide insight into the needs of families with T2D related to online information, while discussing the challenges and potentials of supporting mutual involvement in families with T2D via Web-based health care solutions.

## Methods

To investigate the objectives of this project, we developed a questionnaire to determine the preferences of families with T2D on information content and the presentation of online information on T2D. The inclusion criteria in this study were: Danish families who had access to the Internet, and at least one family member diagnosed with T2D. Participating family members had to be between 15 and 80 years old. Families were excluded if the patient did not include at least one of his/her family members in the survey. Families were recruited between April and May of 2016. The sample size of the study was 50 participants from 22 families with T2D.

### Recruitment Process

The recruitment process of study participants was undertaken with the assistance of 38 HCPs who were identified with help from the Danish Diabetes Association and subsequently contacted by email. These HCPs had previously been involved in the initial phase of the project regarding family needs and problems in relation to family life with T2D, and they were therefore familiar with the scope of the study [22]. Of these 38 HCPs, 9 did not respond and 21 replied that they were interested in recruiting participants for this project. Of the interested HCPs, 6 recruited at least one family with T2D. The occupations of these 6 HCPs were: nurse (2), dietician (2), health consultant (1), and coordinator in a health care facility (1). Each HCP came from a different city across Denmark. The HCPs contacted patients with T2D through consultations, diabetes schools, emails, and phone calls. Patients who were interested in participating in the study were contacted by the person responsible for the project (TV) by email or telephone, and were asked how many relatives were interested in participating in the study. One questionnaire was sent for each participating family member. Patients were excluded if none of their relatives were participating, and relatives were excluded if the family member with T2D withdrew from the study. Nonrespondents were sent a reminder email after 2 weeks, followed by a maximum of two further reminders.

### Questionnaire Design

With no validated instrument to investigate family perceptions of online health care information, a quantitative self-reported questionnaire was developed. The questionnaire was inspired by the work by Jones et al [23] and consisted of 38 questions, including 6 questions on participant characteristics, 5 on preferences in the source of information on T2D, 3 on Internet usage, 12 regarding interest in online information on six problem domains within family life related to T2D, and 10 questions on preferences regarding the presentation of online information and peer-to-peer communication. Questions regarding the participants’ preference in the source of information on T2D were answered by rating 5 choices from 1-5, with lower scores indicating a higher preference. Questions regarding families’ interests and preferences in the six problem domains and in the presentation of online information were answered using a 5-point and a 6-point Likert scale, respectively. These questions were developed based on comprehensive qualitative data from similar settings that focused on the relationships and interactions within families with T2D [22]. The analysis of this study’s results was done with these qualitative findings in mind. Furthermore, the six domains used for this project were identical to the problem domains identified by Grabowski et al [22] and consisted of *Support*, *Knowledge*, *Communication*, *Worries*, *Roles*, and *Everyday Life*. Two questions were asked for each domain. Regarding the presentation of online information, participants were asked to indicate their preference towards information delivered through text and video format, their preference towards information delivered by HCPs and other families with T2D, their interest in references for additional information, and the relevance of providing differentiated information based on the reader (eg, is the reader a patient or a relative). Two open-ended questions were included at the end of the questionnaire to elicit any additional comments or suggestions for improving online information aimed at families with T2D. Answers from these two open-ended questions were, however, excluded from the results due to a lack of relevant answers. The questionnaire was tested with two families prior to the collection of data to ensure that questions were unambiguous and had the right focus. During the data collection phase, the questionnaire was first sent to 10% of the participants to assess data quality. Each family member was instructed to answer the questionnaire individually.

### Statistical Analysis

The results from the questionnaire were transposed from self-completed paper or Word questionnaires into an Excel (version 10; Microsoft for Windows) spreadsheet and SPSS (Version 23; IBM for Macintosh) software for further analysis. Shapiro-Wilk tests were performed to test for normality. Independent t-tests were used when data were normally distributed, and Mann-Whitney U tests were used when data was not normally distributed. Significance was taken at 5% level.

### Ethical Considerations

The Danish Research Ethics Committee has approved the study (reference number H-15006088). All participants gave informed consent.

## Results

Thirty-two patients with T2D were recruited by the HCPs but four did not respond when contacted by the person responsible of the project (TV). One patient withdrew before being included and five patients were excluded for not including their relatives in the study. A total of 22 families were included in the project, incorporating 50 respondents (28 relatives and 22 patients), which corresponded into a response rate of 69%. Seventeen families (17/22, 77%) consisted of the patient and one relative (eg, spouse, parent, offspring, or friend), four families (4/22, 18%) included three family members, and one family (1/22, 5%) included four family members. 12 families answered the questionnaire by letter and 10 families answered by email.

### Demographics of Participants

The group of relatives were mostly male (15/28, 54%), between 50-59 years old (7/28, 25%), and most often a spouse or partner to the patient (18/28, 64%). The group of patients were mostly female (16/22, 73%), between 60-69 years old (11/22, 50%), and had been diagnosed with T2D for less than 10 years (13/22, 59%). Most respondents in each group used the Internet on a daily basis (27/28, 96% vs 20/22, 91%), perceived information on T2D to be relevant (25/28, 89% vs 22/22, 100%), and had a higher education of 2-3 years or a primary school education/equivalent (9/28, 32% vs 8/22, 36%). None of the relatives were diagnosed with T2D. The population characteristics of the relatives and the patients are presented in Table 1 and Table 2, respectively.

**Table 1:** Participant characteristics for relatives.

Characteristics		N=28, n (%)
Females		13 (46)
Usage of the Internet daily		27 (96)
Perceives information on T2D to be relevant		25 (89)
**Education**		
	Primary school, skill in craft or equivalent	9 (32)
	2-3 years of higher education	9 (32)
	3-4 years of higher education	6 (21)
	>4 years of higher education	4 (14)
**Age**		
	Mean age (years)	50
	<30	3 (11)
	30-39	5 (18)
	40-49	4 (14)
	50-59	7 (25)
	60-69	6 (21)
	>70	3 (11)
**Relationship to the patient**		
	Spouse/partner	18 (64)
	Offspring	8 (29)
	Friend	2 (7)

**Table 2:** Participants characteristics for patients.

Characteristics		N=22, n (%)
Females		16 (73)
Usage of the Internet daily		20 (91)
Perceives information on T2D to be relevant		22 (100)
**Education**		
	Primary school, skill in craft or equivalent	8 (36)
	2-3 years of higher education	8 (36)
	3-4 years of higher education	4 (18)
	>4 years of higher education	2 (9)
**Age**		
	Mean age (years)	60
	<30	1 (5)
	30-39	2 (9)
	40-49	1 (5)
	50-59	4 (18)
	0-69	11 (50)
	>70	3 (14)
**Diagnosis**		
	Diabetes duration <10 years	13 (59)
	Mean diabetes duration (years)	9

### Preferences in Access to Diabetes Information

The group of patients clearly indicated a preference for information delivered by HCPs, with a mean score of 1.2 (lower mean scores indicate a higher preference for the source of information on T2D). Most of the patients (18/22, 82%) rated the HCP as their first pick, while the Internet was a clear second pick for the majority of respondents (3/22, 14%; mean score=2.3). The group of relatives were somewhat split between the Internet (11/27, 41%; mean score=2.0) and HCPs (13/27, 48%; mean score=2.2) as their preferred source of information on T2D, which could suggest that relatives do not have the same relationship to HCPs regarding T2D as patients do. The mean difference between the two groups’ preference for information from HCPs resulted in a statistically significant difference (*P*=.006). A lower self-perceived preference for receiving information on T2D through online forums was similar for both groups, along with family and friends, and books (0-7%, mean scores=3.2-4.1). There was, however, a statistically significant difference between the two groups regarding their preference for information from family and friends (*P*=.016) with relatives generally indicating a higher preference than patients (Table 3). A Mann-Whitney U test was performed to assess whether the difference between the groups was statistically significant.

### Usage of the Internet to Search for Information on Type 2 Diabetes

In terms of searching for general online information on T2D, most relatives (16/28, 57%) and patients (19/22, 86%) responded that they had done so, or had others search for information on their behalf. Patients were significantly more likely to have used the Internet to gain information on T2D than relatives (*P*=.027). Despite the relatively large number of participants who had searched for general information on T2D, only a minority of relatives (9/28, 32%) and patients (10/22, 46%) had used the Internet for information regarding how T2D can affect the whole family. Of the relatives and patients who had searched for online information on T2D regarding family life, most indicated that they found what they were looking for (Table 4). A Mann-Whitney U test was performed to assess whether the difference between the groups was statistically significant.

### Interest in Online Information on Type 2 Diabetes Regarding Family Life

**Figure 1:**
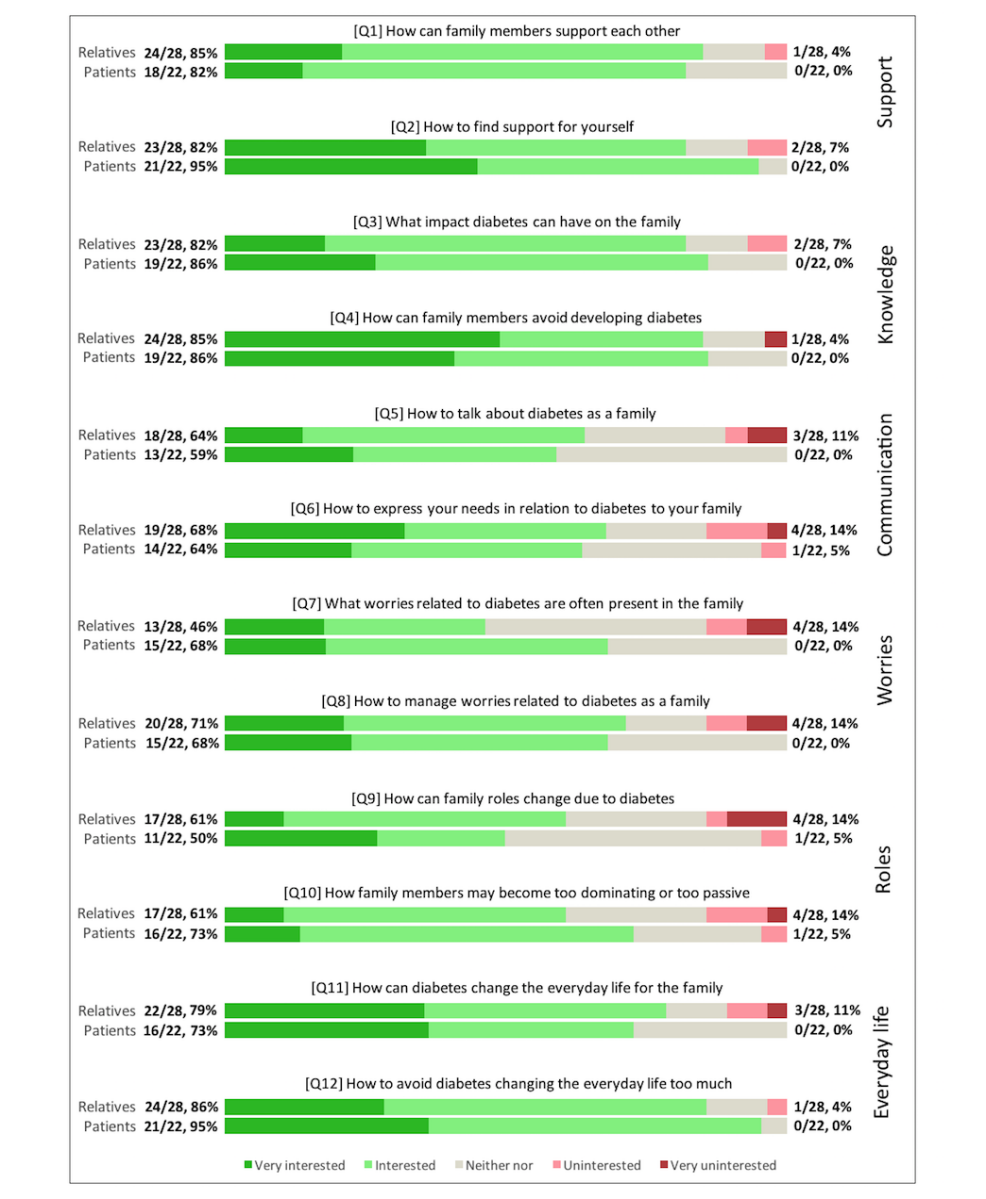
Interest in online information on type 2 diabetes regarding family life for all participants. The absolute values and cumulative percentages of the responses for both groups are displayed at the end of each bar.

Families generally perceived all six domains as relatively interesting although there was a tendency for the three domains of *Support*, *Knowledge,* and *Everyday Life* to be slightly more popular. These three domains were perceived as “Interesting” or “Very interesting” by 73-95% of the patients and by 79-85% of the relatives (Q1-Q4, Q11, and Q12). The remaining three domains (*Communication*, *Worries*, and *Roles*) were perceived as “Interesting” or “Very interesting” by a smaller majority: 50-73% of the patients and 46-71% of the relatives (Q5-Q10). These three domains also received a larger amount of “Neither nor” responses as compared to the other domains, suggesting difficulties in understanding or relating to the questions. The findings may indicate that families perceive information on *Support*, *Knowledge,* and *Everyday Life* as the most relevant and relatable domains. A tendency for all six domains was that the group of relatives more often responded “Uninterested” or “Very Uninterested” to the questions compared to the group of patients. These uninterested responses may partly be explained by the relatives who indicated that information on T2D was irrelevant for them. The questions in Figure 1 were translated and shortened to ease the reading of the figure.

**Table 3:** Preferences in the source of information on T2D

Information source	Relatives (N=27)^a^ mean score (%)	Patients (N=22) mean score (%)	P-value
The Internet (eg, fact-based website)	2.0 (41%)	2.3 (14%)	.319
HCP	2.2 (48%)	1.2 (82%)	.006
Online social forums	3.4 (7%)	3.6 (0%)	.681
Family and friends	3.2 (4%)	4.0 (5%)	.016
Books	4.1 (0%)	3.8 (0%)	.306

^a^One relative made an invalid data entry and was excluded.

**Table 4:** Usage of online information on T2D.

Specifics of search	Relatives (N=28) (%)	Patients (N=22) (%)	P-value
Searched for general online information on T2D	16 (57%)	19 (86%)	.027
Searched for online information on T2D regarding family life	9 (32%)	10 (46%)	.341
Found what they were looking for (only including those who searched for online information on T2D regarding family life	19 (67%)	15 (70%)	.879

**Figure 2:**
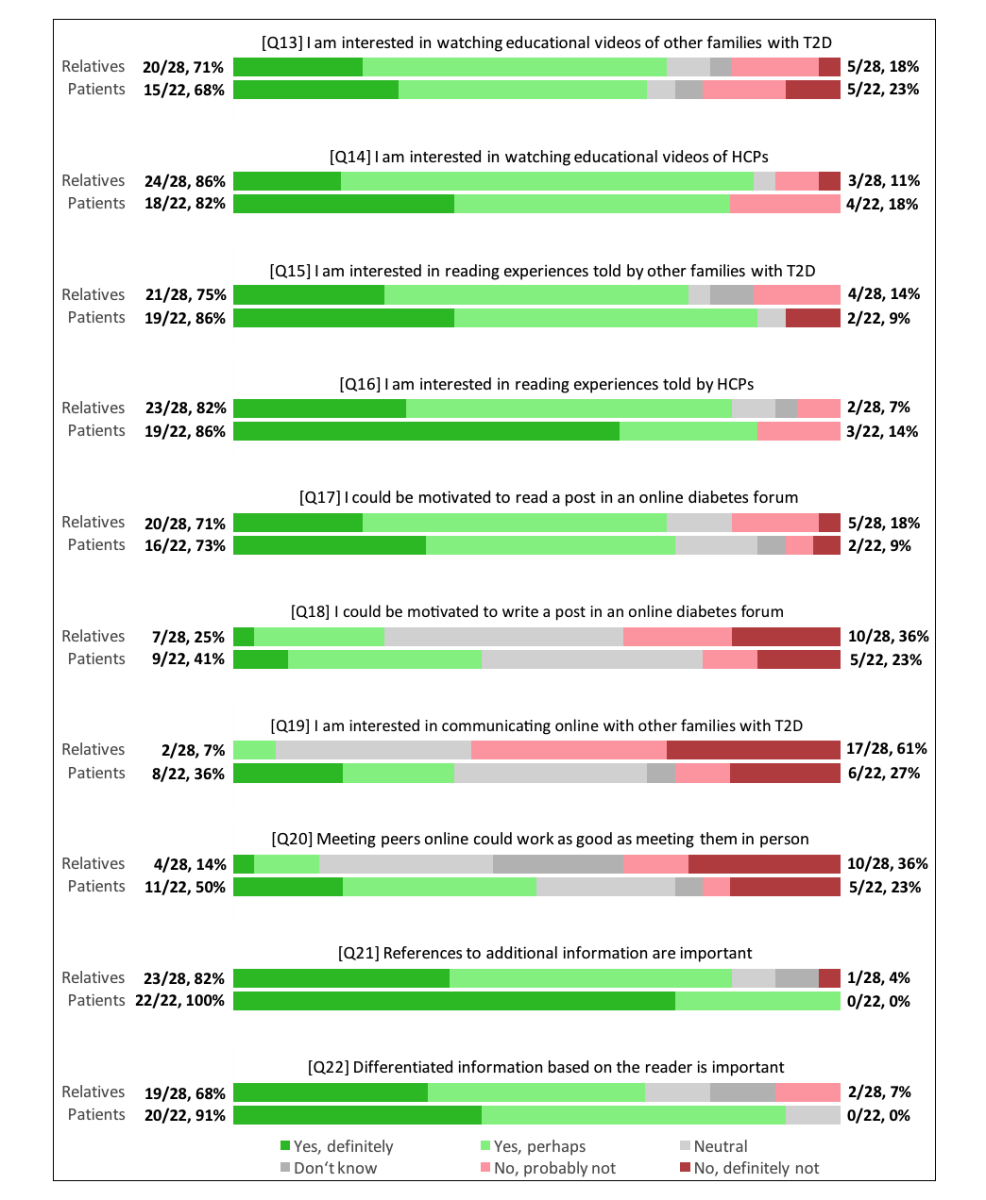
Preferences in the presentation of online information on type 2 diabetes and peer-to-peer communication for all participants. The absolute values and cumulative percentages of the responses for both groups are displayed at the end of each bar. HCP: heath care provider.

A total of 71% of the relatives and 68% of the patients indicated an interest in watching educational videos of peers, while videos of HCPs were considered interesting by 86% and 82% of the relatives and patients respectively (Q13, Q14). Regarding the reading of relevant experiences written by peers, 75% of the relatives and 86% of the patients indicated interest, while 82% and 86% of the relatives and patients were interested in reading experiences by HCPs (Q15, Q16). Patients were more likely to respond “Yes, definitely” for reading experiences by HCPs compared to peers. With regards to communicating with peers online, patients generally responded more positively than relatives. Although 71% of relatives and 73% of patients indicated that they could be interested in reading relevant posts in an online forum (Q17), only 25% of relatives and 41% of patients indicated that they were likely to make a post themselves (Q18). Furthermore, only 7% of relatives and 36% of patients perceived communicating directly with peers as interesting (Q19). Communicating with peers online was considered to be as good as communicating with peers in person by 14% of the relatives, while 50% of the patients thought so (Q20). Overall, the questions on online communication with peers received a considerably higher amount of “Don’t know” and “Neutral” responses, suggesting difficulties in understanding or relating to the questions for many family members. Lastly, 82% of relatives and 100% of patients highlighted the importance of having references to additional information (Q21), while 68% of relatives and 91% of patients indicated a need for information that is differentiated based on whether the reader is a patient or relative (Q22). The questions in Figure 2 were translated and shortened to ease the reading of the figure.

## Discussion

### Families’ Strategies to Find Online Information on Type 2 Diabetes

Overall, most of the patients and relatives in this study indicated that information on T2D was relevant for them and that the Internet was the first or second preferred source when they needed information on T2D. Online information is more accessible and not time dependent compared to a consultation with a HCP, so Web-based solutions could be a relevant and appropriate way for families to gain support and offer an opportunity to establish an appropriate knowledge base on T2D. Relatives were generally less likely to have searched for online information on T2D than patients, suggesting that many relatives either receive information elsewhere (eg, from the patient) or that relatives do not receive (or seek) information on T2D at all (Table 4). Furthermore, there was a discrepancy between the families’ interest for online diabetes information on problem domains within family life and their likelihood of having searched for this information. These findings may illustrate a challenge in involving the whole family in the care of the patient’s T2D, as existing literature has previously concluded [9,10,22]. In addition to this challenge, families may also experience difficulties in locating relevant online information, since much of the educational information on T2D that exists on the Internet has been assessed as insufficient [6,13]. The lack of quality information on the Internet may result in a risk of uncorrected misinformation, misunderstandings, and frustrations for the user [18]. Therefore, since families have indicated an interest in online information on T2D, it would be relevant to ensure that families have easy access to validated, reliable, and user-friendly information on T2D regarding family life.

### Presenting Online Information on Type 2 Diabetes

Overall, the participants in this study indicated a preference for one-way communication (ie, read, watch, or listen to information) compared to two-way communication (ie, communicating with peers or HCPs). Families had a small tendency to prefer educational videos of HCPs over videos of peers, which may suggest that information from HCPs is perceived as more trustworthy than information from other families with T2D. The families’ preferences could also be explained by the concept of the “mere-exposure effect”, suggesting that the increased preference for HCPs as the source of information may be due to families being more familiar with receiving health information from HCPs than from peers [24]. With regards to receiving information from texts or videos, a study by Walthouwer et al [25] found that there are no significant outcome differences between receiving health related text information compared to information presented through videos. However, participants who receive information in their preferred delivery format are significantly more likely to use the information. Therefore, to promote the likelihood of families using online information on T2D, it would be relevant to provide users with information presented through both video and text.

### Online Peer-to-Peer Communication

Online forums were considered to be one of the less preferred routes for receiving information, with patients being more interested in peer-to-peer communication than relatives. Due to the challenges of engaging the whole family in the patient’s T2D, families may not be able to identify the relevance of communicating with other families or be able to assess its benefits. Although most of the families in this study indicated no interest in communicating with peers online, online communities for families with T2D have been shown to be a useful tool for exchanging information and for emotional support [4,5]. One barrier for online forums is that new or potential users of online forums are often cautious and reticent about taking an active role in a forum. This barrier makes it difficult to develop an online community, and its success in the start-up phase is often dependent on subtle prodding from moderators and existing users [26]. A common issue for online communities is a lack of active users, which may weaken the effect of an online community, and as stated by Richardson et al, “size does matter in an online community” [27]. However, if new users become familiar and comfortable in an online community, they tend to become more actively involved and appreciative of the forum over time [26]. Since most families in this study indicated that they would read online posts written by peers–thereby indicating that they would use the online forum as a one-way communication form–it is possible that a professionally moderated online forum could engage motivated families with T2D to communicate with each other. More research regarding family perceptions of online social forums is needed to identify the challenges and potential of online communities for families with T2D.

### Strengths and Limitations of the Study

A major strength of the presented study was its unique focus on the whole family in relation to their needs and preferences, while building on existing evidence regarding the six problem domains for families with T2D. Only a minority of studies have previously focused on mutual involvement, support, and empowerment for the whole family in families with T2D. One strength of the questionnaire survey was its combination of questions on both content and delivery of online information. Although research on user perspectives regarding both content and delivery of online information is uncommon, it appears to be a relevant and appropriate method to gain valuable insight on what content the user is interested in and how the content should be presented [20,21]. A limitation of this study was its sample size and gender imbalance, which questions the statistical power and the lack of knowledge regarding the relatives’ level of engagement in the patient’s disease management. The study does, however, build on recent comprehensive qualitative data and serves as a needs assessment in a significant yet under-researched area. The sample size may also highlight challenges in recruiting families with at least one family member diagnosed with T2D for research projects. Lastly, the study lacks clarity on whether the presented findings might be applicable for different population groups. As identified elsewhere, it will be relevant for future research to investigate whether generic information aimed at families with T2D is sufficient, or if information should be differentiated based on age, gender, ethnicity, or socioeconomic status [28].

Web-based solutions could be a promising tool to inform and support families with T2D by being highly accessible and providing options for differentiated information based on the user’s competences. Since families have indicated an interest in receiving health information through the Internet, improvement of the available online information on T2D regarding families is needed. Findings from this study suggest that information should be presented through both video and text, as families valued both information formats. Online social forums for families with T2D appear to be difficult to develop, but require more research to better understand the potentials and challenges of these platforms.

### Implications for Diabetes Websites

Findings from this study indicate that most families with T2D are interested in using the Internet to gain knowledge on T2D regarding family life. Still, the literature indicates that much of the online information on T2D is insufficient and improvements may be needed to better support families with T2D [6,13]. Previous studies suggest that Web-based solutions aimed at families with a chronic disease could be a supportive instrument for the whole family [29,30]. Therefore, ensuring access to relevant online information of an acceptable quality may be useful for interested families with T2D. Based on findings from the questionnaire, it will be relevant to provide families with online information on six problem domains related to family life with T2D: *Support*, *Knowledge*, *Communication*, *Worries*, *Roles*, and *Everyday Life*. Previous studies have also identified similar domains for families with chronic diseases, while also assessing diet and heredity as popular topics [4,5,31]. With regards to the presentation of online information, families appear to be interested in educational information delivered through text and videos, and relevant experiences told by HCPs and peers, while also indicating that references to additional information is important.

### Implications for Health Care Practice

Previous studies have highlighted the importance of making information appropriate, practical, and accessible for families with T2D. Consequently, considerations on how families become aware of the information are important [32]. Since families have indicated an interest for online information on T2D regarding family life, HCPs are encouraged to refer families to websites with tailored information aimed at families with T2D. However, if the current information on T2D regarding family life is as insufficient as the literature suggests, improvements may be needed before HCPs can refer families to diabetes websites with comprehensive information. In addition, it will be relevant to consider how to approach families of different socioeconomic statuses, who may have problems accessing online information.

### Implications for Future Research

This study has identified preferences and needs for online information in families with T2D, but there is still a need for further studies focusing on online information aimed at families with T2D. With the presented findings on family preferences, it will be relevant to assess whether diabetes websites meet these preferences, while also measuring the quality of the information. Previous research has assessed online information on T2D to be insufficient, and a comparison of the literature suggests that online health educational information on cancer, cardiac diseases, and cardiovascular diseases is of a higher quality than that on T2D [6,13-17]. In addition, previous studies have found that people with low health literacy levels are less likely to use online health information, and that they tend to prefer short concise health information rather than longer and more detailed information [28,33,34]. As found by Mayberry et al [34], future research should investigate the barriers and facilitators for using online information in individuals with different levels of health literacy, while also investigating how family members may support each other in accessing and understanding online health information. Lastly, it will be relevant to investigate the effects of providing families with tailored information on T2D to determine which characteristics of the information have the most positive effects.

## Abbreviations

HCP: health care provider
T2D: type 2 diabetes
